# Supplementary figures and images for: Self-reported negative outcomes of psilocybin users: A quantitative textual analysis
Source: PLoS One. 2020 Feb 21;15(2):e0229067. doi: 10.1371/journal.pone.0229067 (PMC7034876; doi:10.1371/journal.pone.0229067)

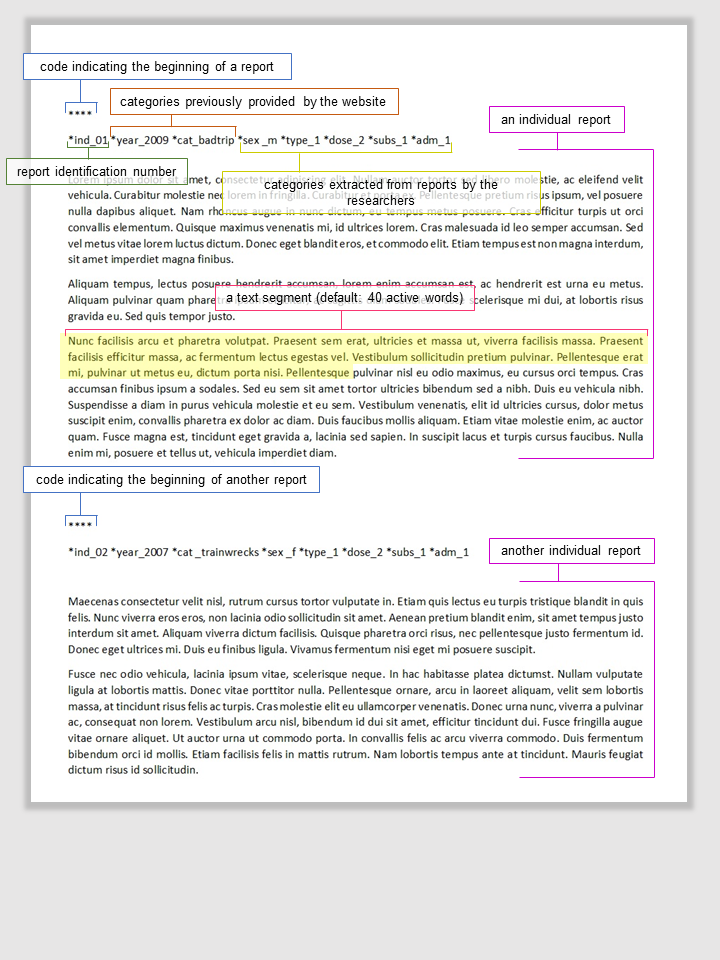

Supplement: S1 Fig — (TIF) [file pone.0229067.s001.tif]
