# Supplementary material for: Self-reported negative outcomes of psilocybin users: A quantitative textual analysis
Source: PLoS One. 2020 Feb 21;15(2):e0229067. doi: 10.1371/journal.pone.0229067 (PMC7034876; doi:10.1371/journal.pone.0229067)
Supplement: Attachment — Submitted filename: Response to reviewers.docx [file pone.0229067.s002.docx]

**Dear editor,**

**Many thanks for your careful handling of our manuscript. We have read the helpful and constructive feedback provided by you and the reviewers and thoroughly revised the paper. Please find below a point-by-point reply to the questions raised by the reviewers.**

Reviewer #1: Comments for Manuscript Number PONE-D-19-25745

This is an interesting study based in an unusual approach which accessed publically available self-reported negative outcomes of psilocybin use. Research on illicit drugs is largely focused on epidemiological data and on etic more than emic perspectives. To understand people`s experiences with psychoactive substances by their own words is absolutely crucial to produce useful knowledge when it comes to design harm reduction strategies or to address possible clinical potential. This is, therefore, a relevant approach. Besides that, the manuscript is clear for both expert and non-expert publics, well organised and well written.

**We thank the reviewer for a thorough reading of the manuscript and positive evaluation of our approach.**

Authors explain the need for that kind of research approaches and sustain what is said in scientific literature. Nevertheless, I think it is possible and desirable to go much further on references used, since this is a hot topic with increasing stiduies available. For example, to address the growing use of psychedelic substances, authors make reference to the Global Drug Survey. This is valid source but there are others (like UNODC or EMCDDA reports) that can be combined with this one. This is even more important if we take in consideration the levels of uncertainty when it comes to prevalence of illicit drug use and to the low control of biases involved in the global drug report collection of data. Besides that, many studies on the effects of psilocybin are now being published, both in controlled and uncontrolled settings.

**We expanded the references used in the literature, encompassing other sources of evidence, such as UNODC and EMCDDA (p. 3).**

The introduction section includes complementary and contradictory data regarding psilocybin potential benefits and harms, which is positive. It also explains how the study will try to contribute to reduce lacunar knowledge in that scenario. A minor comment to that section: at a certain point, authors state that “these sites [devoted to the storage and dissemination of information about psychoactive substances] (…) acting as a valuable resource for prospective or current users”. I tend to agree with this, but we truly don`t know if it is true, unless studies document those positive outcomes…

**We appreciate the reviewers’ concern and we rephrased this slightly, toning down the sentence and emphasizing the opportunity to use self-reported data to detect trends in drug use (p. 4).**

Methods used and correspondent analysis are adequate to an exploratory study (and the fact that there were two researchers performing the analysis is important) and well described. I personally think that a classic content analysis would bring much more insight over what is the aim of the study but, given the exploratory nature of this approach, this research is already a step forward in producing necessary knowledge.

**Although we agree that a content analysis would bring important information, Iramuteq allows calculation of statistical association between text segments with categories and other variables, which was the main purpose of the current submission.**

Minor comments to the method section: authors simply mention that gender of the user will be a variable to analyse, but don´t explain why this was the only socio-demographic element taken in consideration.

**This was done because gender was one of the few socio-demographic variables available from the self-reports. This is now clarified (p. 6).**

However, I see two problems with this methodological strategy:

- one is referred by the authors at the end of the article and refers relevant biases related to the representation of the sample;

- the other one is not mentioned and I think it would be fundamental to take in consideration. Since psilocybin use is classified as illicit, we cannot know, for sure, to what level are those negative outcomes really connected to psilocybin itself or to problems related to the black market vicissitudes (for example not being psilocybin the substance in question). It would be interesting, also, to combine in the conclusion section the results of the study with more scientific literature regarding the same topic, since there are already many studies on the effects of the substance.

**This is an important point, which we now address in the discussion (p. 14). Indeed, the fact that psilocybin is illicit may have an important impact on findings, but, at the same time, our results are representative of current circumstances of use. We also highlighted again in the discussion the relationship between our findings and previous studies (p. 13).**

Reviewer #2: After reviewing this study, I think it is a solid piece of research. There are, however, a few methodological considerations to be made with the suggestion of providing further information in a revised version of the manuscript.

**We thank the reviewer for the careful reading and positive appraisal of the manuscript.**

Before that, I would like to suggest the author that a word of caution should be expressed in the discussion section about the data on which the study is based. They are self-reported reports of the use of psilocybin, and therefore no 'ground truth' about their effective use of substances can be secured. This consideration should be done together with the issue of self-selection

**This is now incorporated in the discussion and mentioned as a potential limitation (p. 14).**

- Having said that, there a few problems with the methodology description that needs clarification:

Reports have been manually categorized using several variables. Some are reporting plain information, while some are more interpretative. Therefore a form of inter-coder reliability should be added since this coding is crucial for the following analysis using Iramuteq. Examples of such coding the appendix would be welcome.

**Thanks for raising this important point. We now include information about the coding and also agreement rates between coders using 10% of the data. Reliability was very high and this additional information has now been included in the manuscript (p. 6-7), with an example of report added as supporting information.**

- The automatic text analysis deployed is sound, and the use of DHA is appropriate giving the context, but some additional information should be provided about what was the unit of context (UC) on which the analysis is performed. Later in the text, the authors mention 'text segments', but it is not clear how these were formally defined. This is an important point as the results of the DHA can vary substantially.

**Text segments are divisions of the text, providing the context in which words appear. Generally, text segments (TS) have three lines, automatically sized according to the corpus extension. In this study we used the default division provided by Iramuteq, with 40 words per text segment. This point is now added to methodology (p.7), with an example of report added as supporting information.**

Information about the average length of the reports should be included.

**This has now been included (p. 6).**

-The outcome of the DHA is expressed in chi-square statistics that reveal the presence of association, in this case between a given set of 'structural variables and linguistic patterns. The relative strength of this association is not part of the output of the software itself, but the authors could test it using phi and creamer's v.

**Thanks for raising this important point. We now report also Cramer’s V (see figure 1 and p. 8 and 9). Considering we reported in figure 1 only the top 25 words associated to each cluster, effect sizes were large or medium.**

If the above points are addressed in a revised version of the manuscript, I believe the paper to be fit for publication.
